# Эндокринный генез развития нарушений менстурального цикла в репродуктивном возрасте

**DOI:** 10.14341/probl13773

**Published:** 2026-05-20

**Authors:** Е. Н. Андреева, Е. В. Шереметьева

**Affiliations:** Национальный медицинский исследовательский центр эндокринологии им. академика И.И. Дедова; Российский университет медицины; Endocrinology Research Centre; Russian University of Medicine; Национальный медицинский исследовательский центр эндокринологии им. академика И.И. Дедова; Endocrinology Research Centre

**Keywords:** нарушения менструального цикла, дисфункция яичников, ожирение, ановуляция, аменорея, бесплодие, гипотиреоз, дидрогестерон, прогестерон, menstrual irregularities, ovarian dysfunction, obesity, anovulation, amenorrhea, infertility, hypothyroidism, dydrogesterone, progesterone

## Abstract

В последние десятилетия правительство РФ поставило задачу по улучшению демографической ситуации в стране. Поэтому врач акушер-гинеколог обращает особое внимание на пациенток репродуктивного возраста, нарушения репродуктивной системы у которых могут приводить к невозможности наступления беременности и развитию бесплодия. Нарушения менструального цикла (НМЦ) — это проявление различных патологических состояний, связанных не только с нарушениями в женской половой системе, но и с общими системными и эндокринными заболеваниями. С 80-х годов XX века частота НМЦ выросла более чем в 7 раз. НМЦ характеризуются изменением цикличности, продолжительности и объема менструальной кровопотери. Врач акушер-гинеколог, к которому первично обращается женщина в репродуктивном периоде с НМЦ, должен не только составить план обследования пациентки, но самое главное — подробно собрать анамнез, включая характеристику физического состояния, спортивный анамнез, пищевые привычки, прием лекарственных препаратов, в том числе витаминов и биологически активных добавок, проанализировать связь НМЦ с эпигенетическими факторами жизни женщины. Частота НМЦ при наличие эндокринного заболевания составляет до 35% и требует направления пациентки к врачу-эндокринологу для компенсации эндокринопатии.

## Abstract (en)

In recent decades, the Russian government has been committed to improving the country's demographic situation. Therefore, obstetricians and gynecologists pay special attention to women of reproductive age, whose reproductive system disorders can lead to infertility and the inability to conceive. Menstrual cycle disorders (MCDs) are a manifestation of various pathological conditions associated not only with disorders of the female reproductive system but also with general systemic and endocrine diseases. Since the 1980s, the incidence of MCDs has increased more than sevenfold. MCDs are characterized by changes in the cycle frequency, duration, and volume of menstrual blood loss. An obstetrician/gynecologist who initially consults a woman of reproductive age with MCDs should not only develop an examination plan for the patient, but most importantly, collect a detailed medical history, including a description of her physical condition, sports history, dietary habits, medication intake, including vitamins and dietary supplements, and analyze the relationship between MCDs and epigenetic factors in the woman's life. The incidence of NMC in the presence of an endocrine disease is up to 35% and requires referral of the patient to an endocrinologist to compensate for the endocrinopathy.

Нарушения менструального цикла (НМЦ) являются одним из частых проявлений гинекологических заболеваний у женщин или могут быть их причиной. Несмотря на значительные адаптационные возможности женского организма, в последние 30 лет отмечен постоянный рост нарушений функции репродуктивной системы [1]. По данным Е.А. Куксиной (2016), число женщин с расстройством менструации с 1980 по 2016 гг. увеличилось в 7,3 раза, средний возраст женщин с НМЦ составляет 29,8 года [2]. Следует подчеркнуть, что данный возраст является «расцветом» репродуктивного периода, когда наши пациентки планируют рождение первого ребенка. Средний возраст матери при рождении первенца в РФ — в среднем 31 год [3]. В последние десятилетия правительство РФ поставило задачу по улучшению демографической ситуации в стране. Поэтому врач акушер-гинеколог должен обращать особое внимание на пациенток репродуктивного возраста, нарушения функции половых органов, у которых могут приводить к невозможности наступления беременности и развитию бесплодия.

Многообразие нозологических форм НМЦ обусловлено его многоступенчатой регуляцией. Развитие НМЦ возможно как вследствие нарушения непосредственно работы гипоталамо-гипофизарно-яичниковой оси (ГГЯО), так и под влиянием на нее извне (например, стресс, нарушение пищевого поведения, некорректно используемая физическая нагрузка и т.д.). Нейрогуморальная регуляция менструального цикла осуществляется в результате согласованной работы коры больших полушарий, определенных отделов гипоталамуса, гипофиза, а также их взаимодействия с эндокринными органами на периферии и рядом внегипоталамических структур. Как правило, НМЦ связаны с изменениями в системе регуляции репродуктивной функции или в органах-мишенях [1].

Несмотря на прогресс современной медицины, в настоящее время проблема эндокринных заболеваний актуальна для большинства стран мира, и Российская Федерация не исключение. В структуре заболеваемости населения эндокринными болезнями лидируют сахарный диабет (СД), болезни щитовидной железы и ожирение. Данные с 2018 г. в отношении структуры заболеваемости эндокринной патологией показали, доля СД составляет 51,2%, заболевания щитовидной железы — 33,7%, ожирение — 14,6% [4]. По данным на февраль 2025 г., две трети россиян имеют одно или несколько эндокринных заболеваний, при этом половина из них еще не знает об этом. Директор Национального медицинского исследовательского центра эндокринологии Минздрава России Наталья Мокрышева отметила, что в настоящее время в России более 5,5 млн пациентов с СД, при этом общее число россиян с этим заболеванием выше, но не все знают о своей болезни [5].

При первичном обращении пациентки с жалобами на нарушения продолжительности и цикличности менструаций необходимо проводить диагностический поиск эндокринной патологии.

Одной из основных проблем ведения женщин в репродуктивном периоде является недостаточная просветительская консультативная работа. Некоторые наши пациентки и их родственники (мамы, бабушки, тетки) не считают НМЦ проблемой и, следовательно, не обращаются за медицинским консультированием при этом состоянии и в отсутствии планирования беременности. Поэтому задачей гинеколога во время консультирования является донести информацию, что регулярный менструальный цикл является маркером физического и психического благополучия у женщин репродуктивного возраста [6].

Общепринятая терминология, к сожалению, не позволяет достичь взаимопонимания специалистов при диагностике и лечении заболевания, которое в МКБ-10 носит название «дисфункция яичников (Е28)». Данный термин по сути является первичным диагнозом, который после осмотра, сбора анамнеза, обследования будет отражать суть и причину НМЦ, следовательно, каждой пациентке будет обеспечен индивидуальный подход к решению ее проблемы.

В 2025 г. были обновлены клинические рекомендации «Аменорея и олигоменорея», в которых экспертная группа определила характеристики нормального регулярного менструального цикла:

- частота (интервал между кровотечениями) — 24–38 дней;

- регулярность (интервалы без кровотечений >20 дней в течение 90-дневного периода) — <20 дней;

- продолжительность кровотечения — до 9 дней [7].

В качестве параметров нормального менструального цикла выступают не только его продолжительность 24–38 дней, но и наличие овуляции и полноценная лютеиновая фаза. При этом продолжительность цикла зависит от длительности фолликулярной фазы, тогда как длительность нормальной лютеиновой фазы — величина относительно постоянная, и должна составлять 14 дней [8].

Частота встречаемости эндокринопатий у женщин репродуктивного возраста была проанализирована на основе профилактических осмотров 11 586 женщин в Волгограде. В структуре первичной заболеваемости (2015) по итогам диспансеризации лидируют болезни эндокринной системы [9].

Синдром гиперпролактинемии. Синдром гиперпролактинемии — это симптомокомплекс, возникающий на фоне гиперпролактинемии, наиболее характерным проявлением которого является нарушение функции репродуктивной системы. Распространенность патологической гиперпролактинемии колеблется от 10 до 30 случаев на 100 тыс. человек, встречается у 5% женщин репродуктивного возраста. Треть женщин на приеме у акушера-гинеколога предъявляют жалобы на различные нарушения менструального цикла, что может быть связано в том числе с повышением уровня пролактина. Согласно статистике, среди женщин репродуктивного возраста с аменореей гиперпролактинемия встречается у 9%, среди женщин с синдромом поликистозных яичников — у 17%. Репродуктологи в РФ должны оценивать уровень пролактина у женщин с бесплодием с целью уточнения генеза ненаступления беременности [12]. Вне зависимости от причины гиперпролактинемии избыточная секреция пролактина приводит к нарушениям пульсаторного выброса ЛГ и ФСГ и, как следствие, к гипогонадизму и бесплодию [10][11]. Дифференциальный диагноз — основа правильной тактики в терапии. Пациентам с гиперпролактинемией рекомендуется: обследование функции щитовидной железы, почек, печени, исключение наличия объемного образования гипоталамо-гипофизарной области, приема ряда лекарственных средств, беременности у женщин [13]. Феномен макропролактинемии — лабораторный феномен, заключающийся в преобладании в образце сыворотки крови высокомолекулярной биологически неактивной фракции пролактина [37]. Макропролактин составляет до 25% гиперпролактинемических сывороток. Макропролактинемия является важной причиной неправильного диагноза, ненужных расследований и «неправильного» лечения. Гормонально-неактивные аденомы, инциденталомы, также могут стать причиной аменореи, поскольку, достигая размеров макроаденом, ухудшают гормональную функцию за счет прямого давления на ножку гипофиза и гипоталамус, вследствие чего развивается вторичный гипогонадизм [14]. Сложность интерпретации показателей базального уровня пролактина обусловлена: транзиторным повышением гормона при стрессах или чрезмерных физических нагрузках, существенной вариабельностью показателей у одного и того же больного при соблюдении всех рекомендаций по сбору крови. При нормализации уровня пролактина, как правило, менструальный цикл стабилизируется, и коррекция не требуется.

Врожденная дисфункция коры надпочечников (ВДКН) или адреногенитальный синдром. Это группа аутосомно-рецессивных заболеваний, характеризующихся дефектом одного из ферментов или транспортных белков, принимающих участие в синтезе кортизола в коре надпочечников. Как правило, врач акушер-гинеколог, наблюдающий взрослое население, встречается на приеме с неклассической формой ВДКН, с более мягким течением и поздним началом (нфВДКН). Неклассическая форма встречается 1:1000 новорожденных [15]. нфВДКН рассматривается как полиэндокринопатия, так как дисбаланс стероидных гормонов не только отражается в нарушениях на уровне надпочечников, но и вызывает изменения в регуляции высших звеньев репродуктивной системы и приводит к развитию ряда нарушений. Диагностику нфВДКН рекомендуется проводить у женщин с признаками гирсутизма, алопеции, акне, нарушениями менструального цикла, бесплодием и/или привычным невынашиванием беременности. Определение утреннего уровня 17ОНР в сыворотке крови в раннюю фолликулярную фазу (не позднее 5–7 дня), при аменорее — в любой день, строго вне беременности. Нормальный показатель 17ОН<6 нмоль/л или <2 нг/мл (ниже этих уровней нфВДКН практически не встречается). Референсные значения, которые приводятся различными лабораториями, обычно отличаются и могут быть значительно ниже указанных «отрезных точек» для диагностики нфВДКН. Дифференциальный диагноз проводят обязательно с СПЯ [15]. Назначения патогенетической терапии при нфВДКН обосновано при хронической ановуляции и отягощенном репродуктивном анамнезе пациентки.

Заболевания щитовидной железы. Заболевания щитовидной железы среди эндокринных нарушений занимают второе место после СХ, причем до 80% из них вызваны хроническим дефицитом йода в питании. Женщины в 10–17 раз чаще, чем мужчины, страдают от заболеваний щитовидной железы. В последние годы распространенность заболеваний щитовидной железы у женщин во время беременности растет, что, несомненно, определяет состояние физического и психического здоровья подрастающего поколения, так как установлено, что даже субклинические формы тиреоидной патологии у матери могут крайне неблагоприятно отразиться на состоянии плода и новорожденного. Патология щитовидной железы может вызвать такие нарушения в репродуктивной системе, как нарушение полового созревания, аменорея, ановуляция, бесплодие, галакторея вследствие гиперпролактинемии, невынашивание беременности [16]. При первичном гипотиреозе нарушения менструального цикла выявлены у 33–80% больных. Первичный гипотиреоз сопровождается нарушениями менструального цикла по типу олиго- или аменореи. Однако ряд исследователей указывают на первичный гипотиреоз как одну из наиболее частых причин обильных менструальных кровотечений [16]. В условиях дисбаланса тиреоидных гормонов изменяется концентрация других стероидных (эстрадиол, тестостерон, кортизол) и гонадотропных гормонов (ЛГ, пролактин), что свидетельствует о нарушении механизмов положительных и отрицательных связей на уровне клеток-мишеней гипоталамуса и гипофиза. У женщин фертильного возраста гипотиреоз приводит к изменениям в длительности цикла и объеме кровопотери в дни менструации, т.е. олигоменорее и аменорее, полименорее и меноррагиям. Дефекты гемостаза, проявляющиеся в снижении уровня факторов VII, VIII, IX и XI, которые наблюдаются при гипотиреозе, также могут способствовать полименорее и меноррагии [14]. Самой частой причиной тиреотоксикоза у молодых женщин репродуктивного возраста является диффузный токсический зоб — диффузный токсический зоб (ДТЗ). Распространенность этого заболевания в популяции составляет 0,1%, у женщин репродуктивного возраста — не менее 0,5%. Действие избытка тиреоидных гормонов на менструальную функцию не является строго специфичным. В работах последних лет частота нарушений менструального цикла значительно ниже, чем в более ранних исследованиях. Это связано, вероятно, с тем, что ДТЗ в настоящее время диагностируется гораздо раньше. Нарушение менструального цикла может быть связано не только с гормональными изменениями, но и с изменением синтеза факторов свертывания крови, например VIII фактора, на фоне тиреотоксикоза или приеме тиреостатиков [17]. При нормализации уровня ТТГ, как правило, менструальный цикл стабилизируется, и коррекция не требуется.

Ожирение. Ожирение — это рецидивирующее полиэтиологическое заболевание. Избыточную массу тела имеют 30–60% женщин репродуктивного возраста, а 25–27% — страдают ожирением. В РФ показатель распространенности ожирения среди женщин — 37,1%. Ожирение у женщин репродуктивного возраста сопровождается высокой частотой ановуляции, синдромом гиперандрогении, нарушениями менструального цикла, патологией эндометрия, бесплодием. При беременности у данной группы женщин выше риск потери на малом сроке, включая беременности в исходе вспомогательных репродуктивных технологий. Ожирение оказывает отрицательное влияние на гипоталамо-гипофизарно-яичниковую ось, нарушает ритм и соотношение гонадотропных гормонов, снижает интенсивность фолликулогенеза и провоцирует снижение уровня прогестерона. Нарушения менструального цикла встречаются чаще у женщин с ожирением и прогрессируют (вплоть до аменореи) при увеличении ИМТ [18]. Большое значение в риске развития нарушений менструального цикла у женщин с избыточной массой тела или ожирением играет не только ИМТ, но и величина окружности талии (ОТ) у женщины. Известно, что женщины репродуктивного возраста с ОТ более 80 см чаще имеют синдром хронической ановуляции в сравнении с теми, у которых такой же ИМТ, но ОТ менее 80 см. Однако следует помнить о женщинах с высоким ИМТ, у которых менструальные циклы регулярные и овуляторные. У таких женщин с течением времени и при отсутствии действий по модификации образа жизни фертильность будет снижаться. Ожирение у женщины репродуктивного возраста может оказывать отрицательное влияние на течение преконцепционного периода, беременности и послеродового этапа [19].

Синдром поликистозных яичников (СПЯ). Это полиэндокринный синдром, характеризующийся нарушением функции яичников (хронической ановуляцией) и гормональной секреции эндокринных желез. Как синдром СПЯ состоит из нескольких важных составляющих компонентов (метаболический, репродуктивный, сердечно-сосудистый, психологический и др.), течение которых формирует патофизиологию заболевания. Основными клинически важными признаками заболевания являются гиперандрогения (клиническая и/или биохимическая) и хроническая ановуляция. Ожирение нельзя назвать обязательным клиническим симптомом СПЯ, однако количество пациенток с ожирением и СПЯ достигает, по данным различных авторов, 45%, что существенно выше, чем в популяции (до 30%). НМЦ встречается, как правило, у всех пациенток с СПЯ. Эта эндокринопатия характеризуется наличием гонадотропной дисфункции, что закономерно приводит к ановуляции или как минимум к недостаточности прогестерона [18][20].

Нарушения углеводного обмена. Патогенетической основой сахарного диабета 2-го типа (СД2) считается ожирение, ассоциированное с инсулинорезистентностью, поэтому и механизмы развития НМЦ аналогичны. Спектр НМЦ у женщин с СД2 характеризуется олигоменореей, полименореей, межменструальными кровянистыми выделениями. Менструации могут становиться не просто частыми, но и длительными, обильными. Иногда они, наоборот, бывают скудными и завершаются в течение 1 сут. Установлено, что продолжительные (более 40 дней) или крайне нерегулярные менструальные циклы могут быть маркером риска развития СД2 [13]. Повышенный риск СД2 сохраняется и у женщин, достигших постменопаузального возраста. Эта ассоциация оказалась сильнее у женщин с избыточной массой тела или ожирением, некачественным питанием и малоподвижным образом жизни [18][21].

Принимая во внимание то, что сахарный диабет 1‑го типа (СД1), имеющий аутоиммунную природу, возникает в раннем возрасте, чаще всего происходит задержка полового развития (пубархе, телархе) c поздним менархе. В дальнейшем развиваются НМЦ по типу олигоменореи или аменореи. Показана более высокая частота олигоменореи (58,9 против 19,6% соответственно) и аменореи (10,7 против 1,8% соответственно) у девочек с СД1 по сравнению с их здоровыми сверстницами. При этом олигоменорея регистрируется у 53,3% девочек с СД1 даже при оптимальном метаболическом контроле [18][22].

В большинстве своем компенсация эндокринопатии приводит к нормализации менстурального цикла, восстановлению овуляции и наступлению беременности при отсутствии других факторов бесплодия в паре.

Но что делать в клинической практике в ситуациях продолжающихся НМЦ у женщин молодого возраста при компенсации эндокринопатии (при ее наличии) и исключении других причин дисфункции яичников (например, стресс-ассоциированные НМЦ), особенно в ситуации планирования беременности?

В 2025 г. Хащенко Е.В. и соавт. провели систематический анализ исследований о влиянии и эффективности и безопасности применения дидрогестерона при лечении нарушений менструального цикла у молодых пациенток, включая период раннего репродуктивного возраста [23]. Обзор подтверждает патогенетическую и клиническую значимость применения дидрогестерона как эффективного и безопасного препарата для терапии нарушений менструального цикла (нерегулярные менструации, аномальные маточные кровотечения, дисменорея) у пациенток репродуктивного возраста, включая ранний репродуктивный период. Прием дидрогестерона сопровождался хорошей переносимостью и высоким уровнем удовлетворенности терапией. Обобщение данных о применении дидрогестерона показало высокую эффективность в регулирующей менструальный цикл терапии, снижении частоты аномальных маточных кровотечений, уменьшении выраженности дисменореи и хронической тазовой боли у пациенток раннего репродуктивного периода. Описаны патогенетические механизмы действия дидрогестерона, включая иммуномодулирующее действие, опосредованное подавлением пути NF-κB/COX-2, синтеза провоспалительных цитокинов и активацией противовоспалительных медиаторов, что способствует восстановлению рецептивности эндометрия, снижению рецидивов аномальных маточных кровотечений и выраженности тазовой боли [23].

Анализ международных данных показывает, что не только на фоне применения дидрогестерона в циклическом режиме, но и далее на фоне отмены препарата у пациенток репродуктивного возраста происходит восстановление менструального цикла. По данным Trivedi N. et al. (2016) [24], на фоне терапии дидрогестероном в дозе 20 мг/сут с 11‑го по 25‑й дни МЦ установился регулярный цикл у 880/910 (96,7%) пациенток с НМЦ, средняя длительность МЦ снизилась на 16,14±24,04 суток, что было охарактеризовано как восстановление нормальной длительности цикла. Из 788 пациенток, доступных для наблюдения, в течение 6-месячного периода после окончания лечения МЦ оставался регулярным у 747 (94,8%) женщин [24]; у 730 (92,6%) не отмечено рецидива, тогда как у 58 (7,4%) рецидив возник в период наблюдения. Зависимости между длительностью лечения в месяцах и стойкостью эффекта (числом месяцев сохранения регулярного цикла) выявлено не было. К тому же снижение средней продолжительности и объема менструального кровотечения сохранялось от момента окончания лечения до окончания периода наблюдения. Wang L. et al. (2020) показали, что регулярный МЦ был достигнут у 72/89 (80,9%) пациенток с АМК, получавших дидрогестерон в дозе 10 мг/сут с 16-го по 25‑й дни МЦ на протяжении 3 МЦ [25]. Таким образом, применение дидрогестерона показало эффективность в восстановлении регулярного МЦ при НМЦ, обеспечивая нормализацию у 80–97% пациенток, с сохранением эффекта после отмены терапии.

Безопасность терапии дидрогестероном оценивали в ряде проанализированных клинических исследований по частоте нежелательных явлений (НЯ) и/или нежелательной лекарственной реакции (НЛР) [23–30]. Во включенных в систематический обзор исследованиях 2025 г. при использовании дидрогестерона зарегистрировали единичные нарушения со стороны репродуктивной системы и молочных желез, психические нарушения (депрессия средней степени тяжести, изменения настроения), тошноту, увеличение массы тела, головную боль. В некоторых исследованиях по изучению дидрогестерона НЯ отсутствовали [23]. В исследовании Kitawaki J. et al. (2021) частота НЯ и НЛР составила 8/59 (13,6%) и 7/59 (11,9 %) соответственно, наиболее частым НЯ было АМК (3/59 [ 5,1%]). Все НЯ разрешились или уменьшилась степень их тяжести [31].

Дидрогестерон in vitro проявляет относительно низкую аффинность к прогестероновым рецепторам, однако in vivo у данного препарата выраженная гестагенная активность, превосходящая натуральный прогестерон. Основной метаболит дидрогестерона, 20α-дигидродигестерон, демонстрирует аналогичную аффинность к прогестероновым рецепторам, при этом его взаимодействие с андрогеновыми и глюкокортикоидными рецепторами выражено в незначительной степени. Высокая рецепторная избирательность дидрогестерона обусловлена жесткой пространственной конформацией, способствующей специфическому связыванию с прогестероновыми рецепторами. Данная молекула представляет собой производное ретропрогестерона — стереоизомера прогестерона с дополнительной двойной связью между атомами углерода С6 и С7. В отличие от почти плоской молекулы прогестерона, ретропрогестерон обладает изогнутой структурой, связанной с изменением пространственного расположения метильной группы в положении С10 и атома водорода в положении С9 [32][33]. Данная модификация увеличивает сродство дидрогестерона к прогестероновым рецепторам и минимизирует связывание с другими рецепторами. В клиническом аспекте дидрогестерон является высокоселективным прогестагеном, активность которого в 10–20 раз выше, чем у прогестерона. Метаболизм дидрогестерона осуществляется путем восстановления кетогруппы в положении С20 с образованием 20α-гидроксипроизводного, а также гидроксилирования метильной группы в положении C21 и атома углерода в положении C16α. Полученные метаболиты сохраняют ретростероидную структуру и фармакологический профиль, схожий с исходным соединением. Минимальное взаимодействие с другими стероидными рецепторами снижает риск побочных эффектов, связанных с активацией андрогенов и глюкокортикостероидов [32]. В некоторых исследованиях продемонстрировано, что дидрогестерон в дозах 10–30 мг обладает схожей способностью в снижении синтеза ДНК в эпителиальных клетках эндометрия и экспрессии ядерных рецепторов к эстрадиолу до уровней, характерных для секреторной фазы менструального цикла, что сопровождается полноценной секреторной трансформацией эндометрия [32].

В настоящее время в РФ представлены несколько препаратов дидрогестерона под разными торговыми наименованиями. В мае 2025 г. зарегистрирован новый препарат «Дидроменс», содержащий микронизированный дидрогестерон в дозе 10 мг («Биннофарм Групп», Россия), полный цикл производства которого находится на международной производственной площадке «Эмкюр Фармасьютикалз» (Индия) [34]. Для оценки фармакокинетики и биоэквивалентности исследуемого лекарственного препарата в 2024 г. было проведено открытое, рандомизированное, репликативное с двумя последовательностями и четырьмя периодами исследование биоэквивалентности препарата «Дидроменс» (Дидрогестерон), таблетки, покрытые пленочной оболочкой, 10 мг (Эмкюр Фармасьютикалс, Индия) и препарата сравнения «Дюфастон®» (Дидрогестерон), таблетки, покрытые пленочной оболочкой, 10 мг (Эбботт Хелскеа Продактс Б.В., Нидерланды).

В исследовании биоэквивалентности участвовали 77 здоровых добровольца, из которых 62 завершили исследование. Продемонстрировано, что препарат «Дидроменс» является биоэквивалентным оригинальному лекарственному препарату с высокой степенью сходства с ним по показателям фармакокинетики, включая концентрацию действующего вещества в плазме крови и относительную биодоступность (таблицы 1 и 2). Изменение концентраций двух лекарственных препаратов в течение времени были сопоставимы. В процессе исследования не было зарегистрировано ни одного серьезного НЯ. На основании результатов проведенных испытаний было показано, что исследуемый лекарственный препарат «Дидроменс», таблетки, покрытые пленочной оболочкой, 10 мг, является биоэквивалентным препарату сравнения таблетки, покрытые пленочной оболочкой, 10 мг, и обладает сопоставимым профилем переносимости и безопасности [34][35]. Это подтверждает обоснованность его применения в клинической практике и в целом повышает доступность терапии дидрогестероном для пациентов.

**Table:** Таблица 1. Полученные фармакокинетические данные (плазма)

Фармакокинетическийпараметр	Арифметическое среднее (± стандартное отклонение)
Исследуемый препарат	Препарат сравнения
AUC (0–t)	8,679 (40,06)	8,485 (43,84)
AUC (0–∞)	10,197 (39,52)	9,971 (44,63)
Cmax	2,732 (47,83)	2,695 (51,59)
tmax	0,98 (62,59)	1,17 (85,01)

**Table:** Таблица 2. Сравнительная таблица фармакокинетических параметров препарата иссл/сравн

Фармакокинетический параметр	Отношение геометрических средних иссл/сравн, %	Доверительные интервалы	CV%
AUC (0–t)	103,49	99,70–107,43	18,39
Cmax	105,48	97,22–114,45	41,48

Прежде чем решать вопрос о «лечении» нарушений менстурального цикла, важно компенсировать состояния, которые могут провоцировать их развитие и формирование прогестерон-дефицита, такие как ожирение, гипотиреоз или гиперпролактинемия (состояния, изменяющие активность оси гипоталамус-гипофиз-яичник). Назначения прогестеронсодержащих препаратов — это в т.ч. эмпирическое лечение на этапе компенсации основной патологии [36]. Нарушение менструального цикла — это не заболевание, а синдром, сопровождающийся комплексом нарушений со стороны женской репродуктивной системы.

## ЗАКЛЮЧЕНИЕ

Этиологические факторы и патогенетические особенности нарушений менструального цикла многообразны. При первичном обращении пациентки с жалобами на нарушения продолжительности и цикличности менструаций необходимо проводить диагностический поиск эндокринной патологии. При выявлении эндокринного заболевания в первую очередь необходимо достичь его компенсации, параллельно восстанавливая нормальный менструальный цикл. Учитывая многофакторность НМЦ при эндокринной патологии, особенно важен мультидисциплинарный подход к ведению пациентки с участием акушера-гинеколога, эндокринолога и терапевта с целью достижения максимальной эффективности и безопасности терапии, а также восстановления репродуктивного потенциала женщины.

## ДОПОЛНИТЕЛЬНАЯ ИНФОРМАЦИЯ

Источники финансирования. Работа выполнена в рамках ГЗ 126022417901-3

Конфликт интересов. Авторы декларируют отсутствие явных и потенциальных конфликтов интересов, связанных с содержанием настоящей статьи.

Участие авторов. Все авторы одобрили финальную версию статьи перед публикацией, выразили согласие нести ответственность за все аспекты работы, подразумевающую надлежащее изучение и решение вопросов, связанных с точностью или добросовестностью любой части работы.
